# Prognostic value of *SRSF2* mutations in patients with de novo myelodysplastic syndromes: A meta-analysis

**DOI:** 10.1371/journal.pone.0185053

**Published:** 2017-09-27

**Authors:** Xue Zheng, Zhi Zhan, Duolan Naren, Jing Li, Tianyou Yan, Yuping Gong

**Affiliations:** 1 Department of Hematology, West China Hospital, Sichuan University, Chengdu, Sichuan Province, China; 2 Department of Cardiology, Zhong Shan Hospital, Fu Dan University, Shanghai, China; 3 Department of Evidence-based Medicine and Clinical Epidemiology, West China Hospital, Sichuan University, Chengdu, Sichuan Province, China; Cardiff University, UNITED KINGDOM

## Abstract

**Background:**

The recent application of gene-sequencing technology has identified many new somatic mutations in patients with myelodysplastic syndromes (MDS). Among them, *serine and arginine rich splicing factor 2* (*SRSF2*) mutations belonging to the RNA splicing pathway were of interest. Many studies have already reported the potential prognostic value of *SRSF2* mutations in MDS patients, with controversial results. Therefore, a meta-analysis was performed to investigate their prognostic impact on MDS.

**Methods:**

Databases, including PubMed, Embase and the Cochrane Library, were searched for relevant studies published up to 14 October 2016. Overall survival (OS) was selected as the primary endpoint, and acute myeloid leukemia (AML) transformation was the secondary endpoint. We extracted the corresponding hazard ratios (HRs) and their 95% confidence intervals (CIs) for OS and AML transformation from multivariate Cox proportional hazards models. The combined HRs with their 95% CIs were calculated using fixed or random effect models.

**Results:**

A total of 10 cohort studies, covering 1864 patients with de novo MDS and 294 patients with *SRSF2* mutations, were included in the final meta-analysis. Our results indicated that *SRSF2* mutations had an adverse prognostic impact on OS (p<0.0001) and AML transformation (p = 0.0005) in the total population. Among the MDS patients with low or intermediate-1 risk defined according to the International Prognostic Scoring System (IPSS), *SRSF2* mutations predicted a shorter OS (p = 0.009) and were more likely to transform to AML (p = 0.007).

**Conclusions:**

This meta-analysis indicates an independent, adverse prognostic impact of *SRSF2* mutations on OS and AML transformation in patients with de novo MDS. This also applies to the subgroup of low- or intermediate-1-IPSS risk MDS. The identification of mutations in *SRSF2* can improve current risk stratification and help make treatment decisions.

## Introduction

Myelodysplastic syndrome (MDS) is a group of clonal hematopoietic stem cell disorders characterized by ineffective hematopoiesis resulting in peripheral blood cytopenias and a risk of progression to acute myeloid leukemia (AML)[1]. The International Prognostic Scoring System (IPSS) and its revision (IPSS-R), depending mainly on bone marrow cytogenetics, marrow blast percentage and the presence of cytopenias, have been widely used as the standard for assessing prognosis and guiding treatment decisions for MDS patients[2,3]. Neither the IPSS nor the IPSS-R considers somatic mutations, though they play a significant role in the diagnosis and prognosis of leukemia. Recently, the application of whole-genome sequencing and whole-exome sequencing has led to the identification of new somatic mutations, which may provide important prognostic information in patients with MDS[4,5].

Through the above techniques, numerous mutations in genes encoding RNA splicing machinery, epigenetic modifiers, chromatin modifiers, transcription factors, signal transducers, RAS pathway members, cohesin complex members and DNA repair proteins have been identified in MDS[6]. Among them, gene mutations involved in the RNA splicing pathway are the most frequent molecular abnormalities[7,8]. RNA splicing is a process that generates mature mRNAs by excising introns and splicing exons from pre-messenger RNA (pre-mRNA); alteration of the excising and splicing of a single pre-mRNA transcript can be used to produce many different mRNAs and result in protein diversity[9,10]. The spliceosome mutations in MDS affect the major components of both the E and A splicing complexes in a mutually exclusive manner, leading to the impaired recognition of 3′-splice site recognition during pre-mRNA processing, inducing abnormally spliced mRNA species and compromising hematopoiesis[5].

One of the potential candidate genes involved in the RNA splicing pathway is *serine and arginine rich splicing factor 2* (*SRSF2*). *SRSF2*, located on chromosome 17q25.2, encodes a protein belonging to the serine/arginine-rich (SR) splicing regulatory factor family[11]. *SRSF2* plays a role in preventing exon skipping, ensuring the accuracy of splicing and regulating alternative pre-mRNA splicing[11]. Although the precise role of *SRSF2* mutations in leukemogenesis remains elusive, many studies have already reported the potential prognostic value of *SRSF2* mutations in MDS patients, with controversial results. Lin et al. [12] and Thol et al. [13] reported *SRSF2* mutations were significantly correlated with poor survival in patients with MDS, while others [14,15] reported no prognostic impact of *SRSF2* mutations. Hence, we performed a meta-analysis on data from related published studies to further explore the combined prognostic impacts of *SRSF2* mutations for patients with de novo MDS.

## Materials and methods

### Literature search

We conducted a literature search of several databases, including PubMed, Embase and the Cochrane Library, for potentially relevant studies published up to October 14, 2016. The following terms were used to find eligible studies: “*SRSF2*” or “*serine and arginine rich splicing factor 2*” or “SC35” and “myelodysplastic syndrome” or “MDS” or “myelodysplasia” or “preleukemia”. No language limitations were added to the search strategy. References of eligible studies were manually searched to find other potentially relevant articles.

### Inclusion and exclusion

Only papers that met all the following criteria were included: (1)The study focused on the prognostic impact of *SRSF2* mutations in de novo MDS patients; (2)The study provided sufficient survival data for patients with *SRSF2* mutations, at least on overall survival (OS); and (3)The study was published as a full article in English. Review articles, case reports and laboratory studies were excluded. If the same or overlapping data was presented in multiple studies, only the most recent or the highest quality study was included.

Two reviewers (Xue Zheng and Zhi Zhan) screened the database and identified the eligible studies, independently. Disagreements were resolved by discussion.

### Data extraction

Two reviewers (Xue Zheng and Duolan Naren) independently extracted relevant information from each eligible study onto a spreadsheet. The data included the first author’s name, year of publication, country of origin, number of patients, age and gender distribution of patients, criteria for classification of MDS, MDS subtype, and the distribution of patients by IPSS classification. We selected OS as the primary endpoint and AML transformation as the secondary endpoint. Endpoints for OS were defined as either deceased (failure) or alive at last follow-up, and the time to AML transformation was calculated beginning from the time the patient entered the trial to the time of AML diagnosis. We extracted the corresponding hazard ratios (HRs) and their 95% confidence intervals (CIs) for OS and AML transformation from multivariate Cox proportional hazards models to evaluate the prognostic impact of *SRSF2*-mutated compared with unmutated patients with MDS. If the published paper did not report the required data for analysis, we contacted the corresponding authors to obtain missing data. Disagreements between reviewers regarding data abstraction were resolved through discussion.

### Quality assessment

Two reviewers (Xue Zheng and Duolan Naren) independently evaluated the methodological quality of each included study. The quality of cohort studies was evaluated by the Newcastle-Ottawa quality assessment scale (NOS). The NOS includes a total of 9 points, with 4 points for selection, 2 points for comparability, and 3 points for exposure or outcome[16]. Cohort studies that scored six or more points were regarded to be of high quality[16]. Disagreements were resolved by discussion.

### Statistical analysis

Review Manager version 5.3 software, following the recommendation of the Cochrane Collaboration (http://tech.cochrane.org/revman/download), was used to calculate the combined survival impact of *SRSF2* mutations. The prognostic effect of *SRSF2* mutations on OS and AML transformation was evaluated by calculation of the combined HRs and their 95% CIs with the generic inverse variance method. The result suggested statistical significance if the 95% CI did not overlap 1. Moreover, *SRSF2* mutations contributed an adverse survival effect compared to unmutated patients when the HR was more than 1. The heterogeneity of the studies was evaluated through the chi-squared test, with significance set at a p-value of less than 0.10. The I^2^ statistic was used to quantify the heterogeneity. An I^2^ value of less than 25% was regarded as low heterogeneity, a value between 25 and 50% indicated moderate heterogeneity, and a value over 50% suggested high heterogeneity[17]. The random effect model was used if high heterogeneity was observed; otherwise, a fixed effect model was used for the meta-analysis.

We used sensitivity analysis and meta-regression analysis regarding the OS of the total population to evaluate the heterogeneity between studies. Sensitivity analysis was used to assess the influence of each study on the stability of the pooled results by sequential omission of one study at a time. Meta-regression analysis was used to evaluate the possible influence of publication year or study country on outcome when there were 10 or more studies including outcome. Funnel plots, Begg’s tests and Egger’s tests were used to screen for potential publication bias regarding the OS of the total population[18,19]. The calculations were carried out in Stata version 12.0 software (Stata Corp, College Station, TX, USA) with a p-value less than 0.05 being considered significant.

## Results

### Study identification and selection

**Fig 1:**
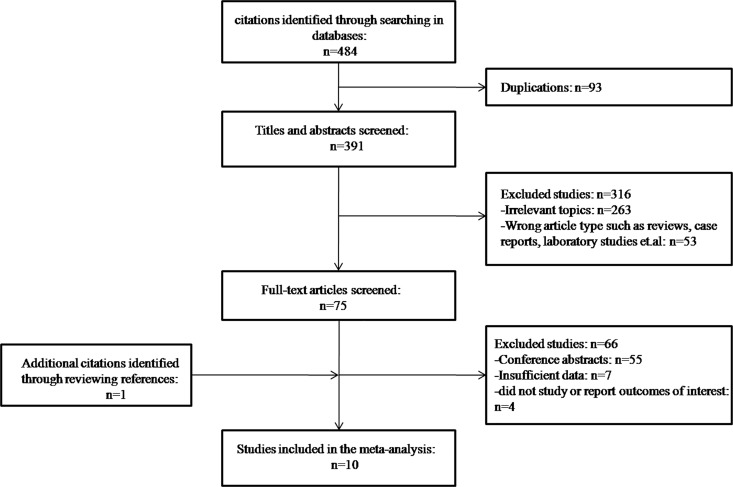
Flow diagram of study selection.

As shown in Fig 1, the initial search revealed 484 studies. After exclusion of 93 duplicates, 391 citations were further reviewed by reading the titles and abstracts, and 316 citations were then excluded for irrelevant article type or irrelevant subject. A total of 75 studies were left for full text review. Among them, 55 studies were excluded as conference abstracts, and 11 were further excluded because they did not evaluate outcome or did not provide sufficient data. During revision, one additional citation was reviewed and included in the final meta-analysis, leaving a total of 10 citations[12–15, 20–25](Fig 1).

### Characteristics of included studies

The 10 included studies were all cohort studies and were published between 2012 and 2016 (Table 1). They were conducted in East Asia, Europe or North America. The studies included a total of 1864 patients with MDS, in which 294 patients harbored a mutation in SRSF2. For one study that included patients with both MDS and AML[23], only patients with MDS were studied in the meta-analysis. Occurrence of mutations of SRSF2 varied between 4.3% and 15% in MDS patients and were more frequent in chronic myelomonocytic leukemia (CMML) (29%-46%).

**Table 1:** Characteristics of studies included in the meta-analysis.

					MDS Subtype					MDS	IPSS		
Study	Country	n/N	Age(years)	Sex(M/F)	RA/RCUD /RARS	RAEB/ RAEB-t	RCMD/ RCMD-RS	CMML	Others	criteria	Low+Int1/Int2+ High/Unknown	Adjustments	NOS
Wu	China	13/304	57(11,89)*	162/142	0/20/9	123/0	0/145	0	7	WHO	212/90/2	1,2,5,6,7	9
2016													
Cui	China	42/145	63(18,85)*	98/47	0/0/0	0/0	0/0	145	0	WHO	NR	NR	8
2015													
Kim	Korea	5/52	52(18,73)*	36/16	2/0/1	27/0	20/0	0	2	WHO	22/30/0	6,7	7
2015													
Kang	Korea	13/129	63.7±12.4#	71/58	0/19/0	46/0	56/0	0	8	WHO	NR	1,2,4,7	8
2015													
Karimi	Sweden	15/100	72(32,88)*	51/49	0/0/10	41/0	2/11	15	21	WHO	NR	1,2,4,7	8
2015													
Lin	China	5/108	60(20,86)*	64/45	10/0/0	48/0	36/8	0	6	WHO	72/32/4	1,2,3,5,7	9
2014													
Itzykson	France	101/312	74(41,93)*	210/102	0/0/0	0/0	0/0	312	0	WHO	NR	7	7
2013													
Thol	Germany	24/193	NR(36,92)*	119/74	38/0/20	53/0	21/9	0	52	WHO	96/51/43	1,3,7	8
2012													
Wu	China	34/233	66(18,95)*	161/72	98	102	0/0	33	0	FAB	115/99/19	1,3,7	8
2012													
Bejar	USA	42/288	69(15,90)*	203/85	173/0/41	71/3※	0/0	0	0	FAB	288/0/0	1,2,3,7	8
2012													

Abbreviations: CMML, chronic myelomonocytic leukemia; FAB, French American British classification; IPSS, International Prognostic Scoring System; MDS, myelodysplastic syndromes; n, number of patients with SRSF2 mutations; N, number of patients in total; NOS, Newcastle-Ottawa quality assessment scale; NR, not reported; RA, refractory anemia; RARS, RA with ringed sideroblasts; RAEB, RA with excess blasts; RAEB-t, RAEB in transformation; RCMD, refractory cytopenia with multilineage dysplasia; RCMD-RS, refractory cytopenia with multilineage dysplasia and ringed sideroblasts; RCUD, refractory cytopenia with unilineage dysplasia; WHO, World Health Organization.

*, median age (range)

#, mean age ± SD

※, the 3 patients with RAEB-t had circulating blasts but≤10% blasts in the bone marrow.

Main adjusted variables in multivariate Cox proportional hazards models: 1 = age; 2 = sex; 3 = IPSS; 4 = IPSS-R; 5 = neutrophils; 6 = hemoglobin; 7 = mutation status

In addition, we managed to contact the authors for some unpublished data. Kim et al. offered the HRs and their 95% CIs for OS after initial treatment of the MDS patients with *SRSF2* mutations in their multivariate cox regression analysis. Similarly, Cui et al. and Bejar et al. also offered the HRs and their 95% CIs for OS in the same setting.

### Quality assessment of included studies

As shown in Table 1, the mean overall NOS score was 8 (range 7–9), indicating that the quality of included studies was high.

### Clinical characteristics of MDS patients with *SRSF2* mutations

Patients carrying *SRSF2* mutations were older than those with unmutated *SRSF2*[12,14,15,21,25], and *SRSF2* mutations were significantly associated with old age (p<0.05)[14,15,21]. Patients with mutations in *SRSF2* had an increased incidence of *RUNX1*, *TET2*, *IDH1*, *IDH2* and *ASXL1* mutations[13,15,21,24].

### Analysis of outcomes

**Fig 2:**
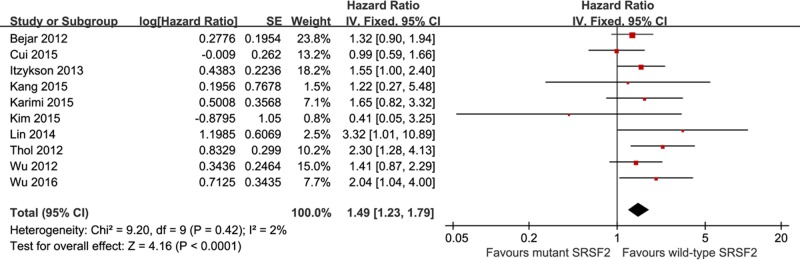
Forest plots of pooled HRs and 95% CIs for OS assessing the prognostic value of *SRSF2* mutations in the cohort of MDS patients.

**Fig 3:**
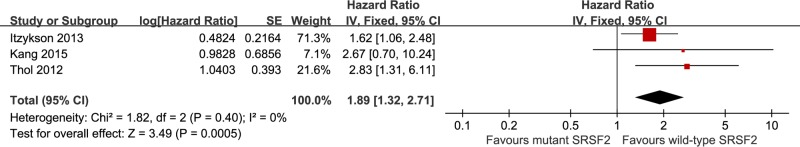
Forest plots of pooled HRs and 95% CIs for AML transformation assessing the prognostic value of *SRSF2* mutations in the cohort of MDS patients.

As shown in Fig 2, the meta-analysis of the effect of *SRSF2* mutations on OS in MDS patients was performed for all cohort studies. Our results indicated that the presence of *SRSF2* mutations was an independent adverse prognostic factor for OS (HR = 1.49, 95% CI: 1.23–1.79, with a p-value less than 0.0001) with a low heterogeneity (I^2^ = 2%). Three studies reported data on AML transformation, with 634 patients and 138 with *SRSF2* mutations (Fig 3). The pooled HR for AML transformation was 1.89 (95% CI: 1.32–2.71, with a p-value of 0.0005, I^2^ = 0%) for *SRSF2*-mutated patients compared with unmutated patients, revealing that patients with *SRSF2* mutations have a more rapid and frequent transformation to AML. There was no heterogeneity among the analyzed studies (I^2^ = 0%).

**Fig 4:**
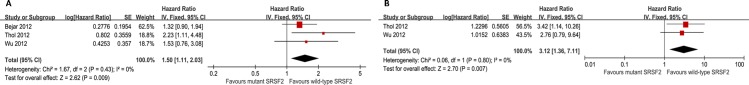
Forest plots of pooled HRs and 95% CIs assessing the prognostic value of *SRSF2* mutations in patients with low- or intermediate-1-IPSS risk MDS for: (A) OS and (B) AML transformation.

Several studies focused on patients with low- or intermediate-1-IPSS risk MDS harboring *SRSF2* mutations. The pooled HR for OS was 1.50 (95% CI: 1.11–2.03, with a p-value of 0.009, I^2^ = 0%), and the HR for AML transformation was 3.12 (95% CI: 1.36–7.11, with a p-value of 0.007, I^2^ = 0%) for patients with low- or intermediate-1-IPSS risk MDS with *SRSF2* mutations, compared to *SRSF2*-unmutated patients (Fig 4), indicating that *SRSF2* mutations predicted an independent unfavorable prognostic impact in terms of both OS and AML transformation. Our results showed no heterogeneity among these studies (I^2^ = 0%).

**Fig 5:**
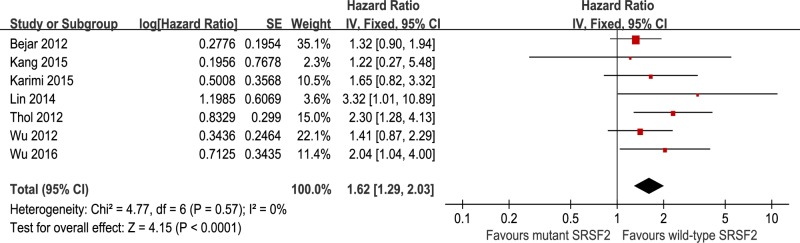
Forest plots of pooled HRs and 95% CIs for OS assessing the prognostic value of *SRSF2* mutations in MDS patients after adjusting for age in COX multivariable models.

Among several studies, *SRSF2* mutations were strongly associated with older age[14,15,21]. We further performed a subgroup analysis, examining whether *SRSF2* mutations could still predict prognosis after adjusting for age. The meta-analysis of *SRSF2* mutations after adjustment for age in COX multivariable models was carried out for 7 cohort studies, with 1355 patients and 146 with *SRSF2* mutations (Fig 5). In this population, patients with *SRSF2* mutations had a significantly shorter OS compared to unmutated patients (HR = 1.62, 95% CI: 1.29–2.03, with a p-value less than 0.0001), with no heterogeneity shown by I^2^ testing (I^2^ = 0%).

### Sensitivity analysis, meta-regression, and publication bias

**Fig 6:**
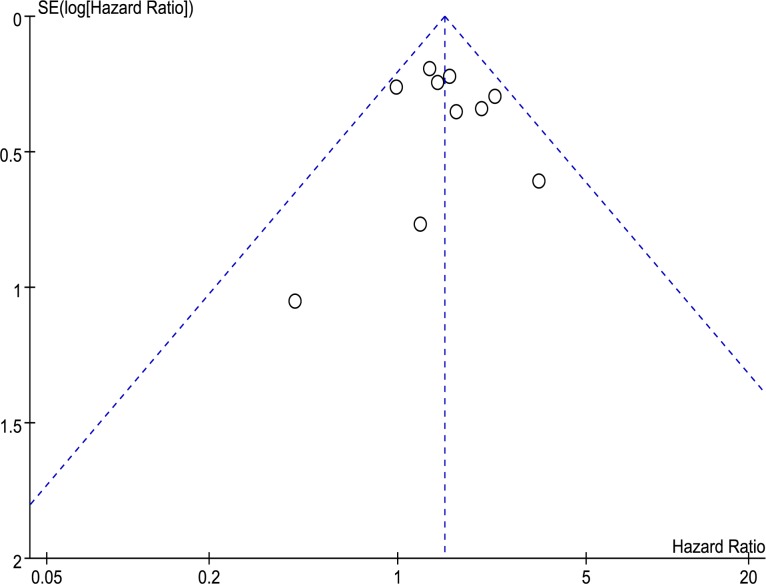
Funnel plots of publication bias for OS of all patients.

We conducted a sensitivity analysis by excluding one study at a time from the meta-analysis, examining the effect of individual studies on the combined HR. The result indicated that there were no significant effects of individual studies on the combined HR for OS in the total population. Meta-regression analysis showed no significant correlations between the publication year or the study country and OS in all patients. There was no significant publication bias in the funnel plot of OS of all population, which contained all 10 studies (Fig 6). The results of Begg’s test and Egger’s test also suggested no publication bias (p = 0.858 of Begg’s test and p = 0.774 of Egger’s test)

## Discussion

The pooled meta-analysis of included studies demonstrated that patients with *SRSF2* mutations had a worse outcome than unmutated patients in the total population, with a shorter OS and a more rapid and frequent transformation to AML. These mutations were also correlated with adverse outcomes in OS and AML transformation in the subgroup of low- or intermediate-1-IPSS risk patients. MDS is a heterogeneous disease with various clinical outcomes, thus, an accurate prediction of prognosis is crucial for selecting the appropriate treatment. Many patients with low- or intermediate-1-IPSS risk MDS have a more aggressive clinical course and an inferior OS than expected. This may be because neither the IPSS nor the IPSS-R takes into account gene mutations, which can provide prognostic information that is not captured by the IPSS and its revision. Our meta-analysis suggested that *SRSF2* mutations were an independent molecular marker for shorter survival and AML transformation in the subgroup of low- or intermediate-1-IPSS risk MDS patients. A combination of *SRSF2* status and IPSS assessment might improve prognostic evaluation for patients with low- or intermediate-1-IPSS risk MDS, and patients with *SRSF2* mutations might benefit from more intensive treatment. However, this analysis is limited by the small number of included studies. More studies concerning *SRSF2* mutations in this subgroup are needed to further verify or modify the results of the pooled analysis.

Increasing age is a well-known significant prognostic factor for survival in MDS patients[3], and our included studies demonstrated that MDS patents with *SRSF2* mutations were of older age compared with unmutated patients. Wu et al. [15] found that the poor prognostic impact of *SRSF2* mutations on OS might be explained by their close association with old age. Our meta-analysis suggested that *SRSF2* mutations still had a significantly poor prognostic effect on OS after adjusting for the effect of age. Thus, mutations in *SRSF2* provide independent prognostic information, which can not be attributed to old age.

Although the precise mechanisms of the contributions of *SRSF2* mutations to the pathogenesis of MDS remain largely unknown, a growing number of studies have explained the mechanisms of *SRSF2* mutations leading to the poor prognosis of MDS patients: (1) *SRSF2* mutations occur early and are implicated as founder mutations in MDS[8,26], suggesting that they may play an important role in disease initiation. (2) *SRSF2* mutations alter normal sequence-specific RNA binding activity, therefore altering the recognition of specific exonic splicing enhancer motifs to drive recurrent mis-splicing of key hematopoietic regulators[27]. Evidence from a study using an *SRSF2* knock-in mouse model demonstrates that *SRSF2* mutations can impair differentiation and increase apoptosis, resulting in peripheral cytopenias and morphologic dysplasia[27]. (3) *SRSF2* has a critical role in maintaining genomic stability and regulating cell proliferation[28]. Depletion of *SRSF2* can cause genomic instability, which may be a potential mechanism for acquiring other gene mutations during disease progression to induce AML transformation [28]. Yoshida et al. reported that approximately 20% of MDS cases have no known genetic changes, thus, *SRSF2* mutations can not fully account for the pathogenesis of MDS[5]. Apart from the studies showing the above mechanisms, further studies are needed to sufficiently explain the contribution of *SRSF2* mutations to MDS.

Our paper is the first meta-analysis concerning the controversial prognostic value of *SRSF2* mutations in MDS patients. However, several limitations should be acknowledged. First, the adjusted factors from multivariate Cox proportional hazard models in each eligible study were different, which might represent methodological heterogeneity among the 10 studies. Second, there was much clinical heterogeneity between studies, such as the diverse treatment programs, age and gender distribution of patients, MDS subtype, cytogenetic and molecular abnormalities, time of follow-up, and the methods of gene sequencing, and this heterogeneity potentially effected clinical outcomes. Third, as *SRSF2* mutations coexist with other mutations, such as mutations of *RUNX1*, *TET2*, *IDH1*, *IDH2* and *ASXL1*, we could not evaluate the potential effects of gene-gene interactions on survival outcome. Finally, although Begg’s test and Egger’s test results suggested that no publication bias was observed, it still existed due to our using only published articles.

In conclusion, our meta-analysis indicates an independent, adverse prognostic impact of *SRSF2* mutations on OS and AML transformation in patients with de novo MDS. This also applies to the subgroup of low- or intermediate-1-IPSS risk MDS. In addition, the inferior OS of patients with *SRSF2* mutations could not be attributed to the close association of the mutation with old age. Mutations in *SRSF2* can provide prognostic information that is not captured by the IPSS and might improve current risk stratification and treatment decisions. A better understanding of splicing mutations is leading to the development of spliceosome inhibitors, which could provide novel targeted therapies for patients[29]. Further studies using prospective randomized controlled trials are needed to confirm the prognostic impact of *SRSF2* mutations, especially in patients with low- or intermediate-1-IPSS risk MDS.

## Supporting information

S1 Checklist PRISMA checklist. (PDF)

S1 Protocol The protocol registered on the PROSPERO. (PDF)
