# Supplementary material for: Prognostic value of SRSF2 mutations in patients with de novo myelodysplastic syndromes: A meta-analysis
Source: PLoS One. 2017 Sep 27;12(9):e0185053. doi: 10.1371/journal.pone.0185053 (PMC5617234; doi:10.1371/journal.pone.0185053)
Supplement: S1 Protocol — (PDF) [file pone.0185053.s002.pdf]

PROSPERO International prospective register of systematic reviews

Review title and timescale

1 Review title

Give the working title of the review. This must be in English. Ideally it should state succinctly the interventions or exposures being reviewed and the associated health or social problem being addressed in the review.

Prognostic Value of SRSF2 Mutations in Patients with De Novo Myelodysplastic Syndromes: A Systematic Review and Meta-analysis

2 Original language title

For reviews in languages other than English, this field should be used to enter the title in the language of the review. This will be displayed together with the English language title.

3 Anticipated or actual start date

Give the date when the systematic review commenced, or is expected to commence.

09/11/2016

4 Anticipated completion date

Give the date by which the review is expected to be completed.

31/12/2016

5 Stage of review at time of this submission

Indicate the stage of progress of the review by ticking the relevant boxes. Reviews that have progressed beyond the point of completing data extraction at the time of initial registration are not eligible for inclusion in PROSPERO. This field should be updated when any amendments are made to a published record.

The review has not yet started ×

Review stage	Started	Completed
Preliminary searches	Yes	Yes
Piloting of the study selection process	Yes	Yes
Formal screening of search results against eligibility criteria	Yes	Yes
Data extraction	Yes	Yes
Risk of bias (quality) assessment	Yes	Yes
Data analysis	Yes	Yes

Provide any other relevant information about the stage of the review here.

Review team details

6 Named contact

The named contact acts as the guarantor for the accuracy of the information presented in the register record.

Ms Zheng

7 Named contact email

Enter the electronic mail address of the named contact.

zhengxueok@163.com

8 Named contact address

Enter the full postal address for the named contact.

Department of Hematology, West China Hospital, Sichuan University, Chengdu, China.

9 Named contact phone number

Enter the telephone number for the named contact, including international dialing code.

86-18782958218

10 Organisational affiliation of the review

Full title of the organisational affiliations for this review, and website address if available. This field may be completed as 'None' if the review is not affiliated to any organisation.

West China Hospital, Sichuan University, China.

Website address:
http://www.cd120.com/

11 Review team members and their organisational affiliations
Give the title, first name and last name of all members of the team working directly on the review. Give the organisational affiliations of each member of the review team.

Title	First name	Last name	Affiliation
Ms	Xue	Zheng	West China Hospital, Sichuan University, China.
Mr	Zhi	Zhan	Zhongshan Hospital, Fudan University, China.
Professor	YuPing	Gong	West China Hospital, Sichuan University, China.
Professor	Jing	Li	West China Hospital, Sichuan University, China.
Ms	DuoLan	NaRen	West China Hospital, Sichuan University, China.

12 Funding sources/sponsors
Give details of the individuals, organizations, groups or other legal entities who take responsibility for initiating, managing, sponsoring and/or financing the review. Any unique identification numbers assigned to the review by the individuals or bodies listed should be included.
The work was supported by the Foundation of the Science & Technology Department of Sichuan Province (No. 2015SZ0234-5), Foundation of Administration of traditional Chinese medicine of Sichuan Province (No. 2014A038) and the Foundation of Science and Technology Bureau of Chengdu (No. 2016-HM01-00001-SF).

13 Conflicts of interest
List any conditions that could lead to actual or perceived undue influence on judgements concerning the main topic investigated in the review.
Are there any actual or potential conflicts of interest?
None known

14 Collaborators
Give the name, affiliation and role of any individuals or organisations who are working on the review but who are not listed as review team members.

Title	First name	Last name	Organisation details
Mr	TIANYOU	YAN	West China Hospital, Sichuan University, China.

Review methods

15 Review question(s)
State the question(s) to be addressed / review objectives. Please complete a separate box for each question.
P: patients with de novo myelodysplastic syndromes.

I: SRSF2 mutations.

C: wild-type SRSF2.

O: overall survival.

S: cohort studies.

16 Searches
Give details of the sources to be searched, and any restrictions (e.g. language or publication period). The full search strategy is not required, but may be supplied as a link or attachment.

We will search the following databases: PubMed, EMBASE, The Cochrane Library. Studies published up to 14 October 2016 will be sought using the search terms "(SRSF2 OR Serine and arginine rich splicing factor 2 OR SC35) AND (myelodysplastic syndrome OR MDS OR myelodysplasia OR preleukemia)".

17 URL to search strategy

If you have one, give the link to your search strategy here. Alternatively you can e-mail this to PROSPERO and we will store and link to it.

I give permission for this file to be made publicly available
No

18 Condition or domain being studied

Give a short description of the disease, condition or healthcare domain being studied. This could include health and wellbeing outcomes.
Patients with de novo myelodysplastic syndromes.

19 Participants/population

Give summary criteria for the participants or populations being studied by the review. The preferred format includes details of both inclusion and exclusion criteria.
Inclusion: Patients with de novo myelodysplastic syndromes (as diagnosed according to WHO or FAB criteria).
Exclusion: therapy-related MDS and AML transformed from MDS.

20 Intervention(s), exposure(s)

Give full and clear descriptions of the nature of the interventions or the exposures to be reviewed
Patients with SRSF2 mutations when diagnosed with de novo myelodysplastic syndromes.

21 Comparator(s)/control

Where relevant, give details of the alternatives against which the main subject/topic of the review will be compared (e.g. another intervention or a non-exposed control group).
Patients with wild-type SRSF2 when diagnosed with de novo myelodysplastic syndromes.

22 Types of study to be included

Give details of the study designs to be included in the review. If there are no restrictions on the types of study design eligible for inclusion, this should be stated.
We will include cohort studies

23 Context

Give summary details of the setting and other relevant characteristics which help define the inclusion or exclusion criteria.
Laboratory articles, reviews and case reports are excluded. The studies should be published as a full-text document in English.

24 Primary outcome(s)

Give the most important outcomes.
Hazard ratios of overall survival.

Give information on timing and effect measures, as appropriate.
The Kaplan–Meier test was used for univariate survival analysis and differences were assessed by log-rank analysis. Multivariate Cox proportional hazards models were used to calculate hazard ratios and 95 % confidence intervals of the associations between risk factors and survival.

25 Secondary outcomes

List any additional outcomes that will be addressed. If there are no secondary outcomes enter None.
Hazard ratios of AML transformation

Give information on timing and effect measures, as appropriate.
The Kaplan–Meier test was used for univariate survival analysis and differences were assessed by log-rank analysis. Multivariate Cox proportional hazards models were used to calculate hazard ratios and 95 % confidence intervals of the associations between risk factors and AML transformation.

26 Data extraction (selection and coding)

Give the procedure for selecting studies for the review and extracting data, including the number of researchers involved and how discrepancies will be resolved. List the data to be extracted.
Titles and abstracts of studies will be retrieved using the search strategy and those from additional sources will be screened independently by two review authors to identify studies that potentially meet the inclusion criteria. The full text of these potentially eligible studies will be retrieved and independently assessed for eligibility by two review team members. Disagreements will be resolved by discussion. Baseline characteristics for each study will be summarized on a spreadsheet. Extracted information will include: first author's name, year of publication, country of origin, participant gender, participant age, sample size, MDS subtype, criteria for classification of MDS and IPSS classification. Two review authors will extract data independently, discrepancies will be identified and resolved through discussion. Missing data will be requested by contacting authors.

27 Risk of bias (quality) assessment
State whether and how risk of bias will be assessed, how the quality of individual studies will be assessed, and whether and how this will influence the planned synthesis.
Possible publication bias will be detected with Begg's and Egger's tests. Two authors will independently assess the methodological quality of each individual study using the Newcastle-Ottawa quality assessment scale (NOS) for cohort studies

28 Strategy for data synthesis
Give the planned general approach to be used, for example whether the data to be used will be aggregate or at the level of individual participants, and whether a quantitative or narrative (descriptive) synthesis is planned. Where appropriate a brief outline of analytic approach should be given.
HRs and their 95% CIs of OS will be used to evaluate the prognostic impact of SRSF2 mutations compared with wild type in patients with MDS. The statistical heterogeneity of the effect will be assessed by the I-squared and Q statistics; we will judge that heterogeneity is significant when the p - value is less than 0.1. A fixed-effect model analysis will be performed when I-squared is less than 50% for the Q test, otherwise a random-effects model will be conducted.

29 Analysis of subgroups or subsets
Give any planned exploration of subgroups or subsets within the review. 'None planned' is a valid response if no subgroup analyses are planned.
The International Prognostic Scoring System (IPSS).

Review general information

30 Type and method of review
Select the type of review and the review method from the drop down list.
Meta-analysis, Prognostic, Systematic review

31 Language
Select the language(s) in which the review is being written and will be made available, from the drop down list. Use the control key to select more than one language.
English

Will a summary/abstract be made available in English?
Yes

32 Country
Select the country in which the review is being carried out from the drop down list. For multi-national collaborations select all the countries involved. Use the control key to select more than one country.
China

33 Other registration details
Give the name of any organisation where the systematic review title or protocol is registered together with any unique identification number assigned. If extracted data will be stored and made available through a repository such as the Systematic Review Data Repository (SRDR), details and a link should be included here.

34 Reference and/or URL for published protocol
Give the citation for the published protocol, if there is one.
Give the link to the published protocol, if there is one. This may be to an external site or to a protocol deposited with CRD in pdf format.

I give permission for this file to be made publicly available
No

35 Dissemination plans
Give brief details of plans for communicating essential messages from the review to the appropriate audiences.
In addition to producing a report for the funders of this review, which will be made available free of charge on their website, a paper will be submitted to a leading journal in this field. Furthermore, we hope to identify the prognostic value of SRSF2 mutations in MDS.

Do you intend to publish the review on completion?
Yes

36 Keywords
Give words or phrases that best describe the review. (One word per box, create a new box for each term)
myelodysplastic syndrome

SRSF2

mutation

prognosis

meta-analysis

37 Details of any existing review of the same topic by the same authors
Give details of earlier versions of the systematic review if an update of an existing review is being registered, including full bibliographic reference if possible.

38 Current review status
Review status should be updated when the review is completed and when it is published.
Ongoing

39 Any additional information
Provide any further information the review team consider relevant to the registration of the review.
This review is being undertaken to indicate the SRSF2 mutations on prognosis for patients with MDS.

40 Details of final report/publication(s)
This field should be left empty until details of the completed review are available.
Give the full citation for the final report or publication of the systematic review.
Give the URL where available.
